# A quantitative framework for patient‐specific collision detection in proton therapy

**DOI:** 10.1002/acm2.14247

**Published:** 2023-12-22

**Authors:** Stephen K. Northway, Bailey M. Vallejo, Lawrence Liu, Emily E. Hansen, Shikui Tang, Dennis Mah, Iain J. MacEwan, James J. Urbanic, Chang Chang

**Affiliations:** ^1^ Department of Radiation Medicine and Applied Sciences University of California at San Diego La Jolla California USA; ^2^ California Protons Cancer Therapy Center San Diego California USA; ^3^ Texas Center for Proton Therapy Irving Texas USA; ^4^ ProCure Proton Therapy Center Somerset New Jersey USA

**Keywords:** collision detection, lateral penumbra, proton therapy

## Abstract

**Background:**

Beam modifying accessories for proton therapy often need to be placed in close proximity of the patient for optimal dosimetry. However, proton treatment units are larger in size and as a result the planned treatment geometry may not be achievable due to collisions with the patient. A framework that can accurately simulate proton treatment geometry is desired.

**Purpose:**

A quantitative framework was developed to model patient‐specific proton treatment geometry, minimize air gap, and avoid collisions.

**Methods:**

The patient's external contour is converted into the International Electrotechnique Commission (IEC) gantry coordinates following the patient's orientation and each beam's gantry and table angles. All snout components are modeled by three‐dimensional (3D) geometric shapes such as columns, cuboids, and frustums. Beam‐specific parameters such as isocenter coordinates, snout type and extension are used to determine if any point on the external contour protrudes into the various snout components. A 3D graphical user interface is also provided to the planner to visualize the treatment geometry. In case of a collision, the framework's analytic algorithm quantifies the maximum protrusion of the external contour into the snout components. Without a collision, the framework quantifies the minimum distance of the external contour from the snout components and renders a warning if such distance is less than 5 cm.

**Results:**

Three different snout designs are modeled. Examples of potential collision and its aversion by snout retraction are demonstrated. Different patient orientations, including a sitting treatment position, as well as treatment plans with multiple isocenters, are successfully modeled in the framework. Finally, the dosimetric advantage of reduced air gap enabled by this framework is demonstrated by comparing plans with standard and reduced air gaps.

**Conclusion:**

Implementation of this framework reduces incidence of collisions in the treatment room. In addition, it enables the planners to minimize the air gap and achieve better plan dosimetry.

## 1 INTRODUCTION

Proton therapy utilizes charged particles’ characteristics dose deposition property, namely the Bragg peak, to deliver ionizing radiation to the tumor.^1^, ^2^ The protons are accelerated to specific kinetic energies corresponding to the depth of the tumor before being emitted into the patient for cancer treatment. The majority of the particle's kinetic energy is deposited around the Bragg peak inside the tumor, leaving no exit dose. This absence of exit dose gives proton therapy one extra dimension in controlling its dose distributions. The superior dosimetry provided by proton therapy can potentially translate into favorable clinical outcomes.^3^, ^4^, ^5^

Proton therapy uses a different set of equipment from conventional photon radiation therapy. Cyclotrons or synchrotrons are used to accelerate the protons.^6^, ^7^ Radiation transport apparatus downstream of the accelerators are also different, for example, large magnets are needed to steer and shape the beam, and 2D monitor‐unit counters are located immediately before the protons enter the patient. As a result, a proton treatment unit's nozzle (the equivalent of linac's treatment head) is typically larger than its photon counterpart. In addition, beam modifying accessories such as range shifters and apertures are often required for proton therapy. These accessories are typically affixed onto a movable snout attached at the end of the nozzle, and are therefore in the proximity of the patient during treatment.

Snout designs vary between different proton equipment vendors and different delivery modalities such as pencil‐beam scanning (PBS) and double‐scattering (DS). Nevertheless, the primary functionality of the snout remains the same, that is, to provide a movable assembly for beam‐modifying accessories. A typical PBS treatment unit's snout contains mounting mechanisms for range shifters to be inserted when treating shallower tumors. The range shifter then moves axially along the beam direction with the snout. Additional beam‐modifying accessories, such as apertures, compensators, and multi‐leaf collimators,^8^, ^9^, ^10^ can also be housed in the snout, although range shifters are the most common and often closest to the patient during treatment.

The air gap between the range shifter and the patient is an important factor that affects the lateral penumbra of the beam.^11^, ^12^ The insertion of a range shifter alters the angular distribution of the proton beam due to multiple Coulomb scattering.^13^, ^14^, ^15^, ^16^ This increased angular distribution at the exit of the range shifter causes the proton spots to expand as they propagate through the air. As a result, larger air gaps translate to wider lateral penumbra. To minimize this scattering effect, it is therefore desirable for proton treatment plans to have smaller air gaps for better dosimetry. However, reducing the air gap increases the likelihood of collision between the proton treatment unit and the patient. Note that here collisions are defined as circumstances where the snout cannot be extended to the planned location due to obstruction by the patient, the treatment table, or the immobilization device. Such unattainable geometries are typically discovered on the first day of treatment and in most cases the treatment plan must be re‐calculated to determine if the lateral penumbra is still dosimetrically acceptable under the actual (i.e., larger) air gap. Often the treatment plan needs to be further optimized to bring in the penumbra, causing delays in the patient's treatments. Therefore, a collision detection program capable of accurately modeling the treatment geometry can also prevent these treatment delays in the clinic.

Numerous collision detection frameworks using different methodologies have been reported for both photon and proton external beam therapy.^17^, ^18^, ^19^, ^20^, ^21^, ^22^, ^23^, ^24^, ^25^, ^26^, ^27^, ^28^, ^29^, ^30^ In general, the treatment head (gantry) and the patient support (table or chair) are modeled with varying degrees of details, that is, from a simple set of connected vertices to actual computer‐aided design files.^18^, ^26^, ^27^, ^31^ The patient has also been represented by a simple geometry like an ellipse^17^ or a column,^32^, ^33^ or skipped for simplicity.^18^, ^19^, ^23^, ^28^ Some frameworks use a phantom or an averaged patient to establish a lookup table of “collision zones” to be excluded when planning.^21^, ^27^, ^34^, ^35^ Patient‐specific contours, generated either by computed tomography (CT) or surface imaging cameras,^27^, ^32^, ^35^, ^36^, ^37^ have also been used where sophisticated numerical methods such as ray‐tracing or mesh triangulations are required for collision detection.^22^, ^26^, ^36^, ^37^, ^38^

Here we present a general quantitative framework to analytically model the geometries between the proton treatment unit and the patient for various snout accessories across multiple vendors. The selection of components to be included in this framework is based on our clinical experience. Specifically, this model takes into account the patient contour, gantry rotation, table angle, snout extension, range shifter thickness, and the dimensions of other vendor‐specific accessories that have previously caused collision in our clinics. Automatic collision detection analytically determines the minimum clearance between the patient and the various snout accessories. When the framework detects a collision, it is represented by a negative value that specifies the exact amount of snout retraction needed to avert the collision. The accuracy of the model is validated using a phantom to be around 8 mm. As a precaution, the framework alerts planners whenever the minimum clearance for a beam is less than 5 cm. This analytic model allows for quantitative and automatic collision detection in about 0.16 s on average (not including the read time for the Digital Imaging and Communications in Medicine (DICOM) structure set and plan), thus can potentially be extended to accommodate proton arc therapy.^39^, ^40^ We have implemented this model in multiple clinics with different snout designs and have successfully reduced the occurrences of collisions in our clinics. Extension of this framework to include other snout designs is straightforward.

## 2 MATERIALS AND METHODS

All plan parameters needed for collision detection, such as gantry angles, couch angles, isocenter coordinates, snout sizes, snout positions, range shifter dimensions and so forth, are extracted from the treatment plan for each beam. Dimensions of the treatment unit and its snout accessories are measured directly. The shape of the patient is modeled by the external contour. For most treatment planning systems (TPSs) in proton therapy, patient support structures (e.g., treatment table) and immobilization devices must also be included into the external contour for dose calculation. As a result, the external contour is readily available here for collision detection. For TPS that use a separate patient support structure, inclusion of such structure into the overall external contour is trivial. Hereafter the terms ‘external’ and ‘patient’ contours will be used interchangeably with the understanding that a collision with the proton treatment unit is between either the patient, the table, or the immobilization devices.

**FIGURE 1:**
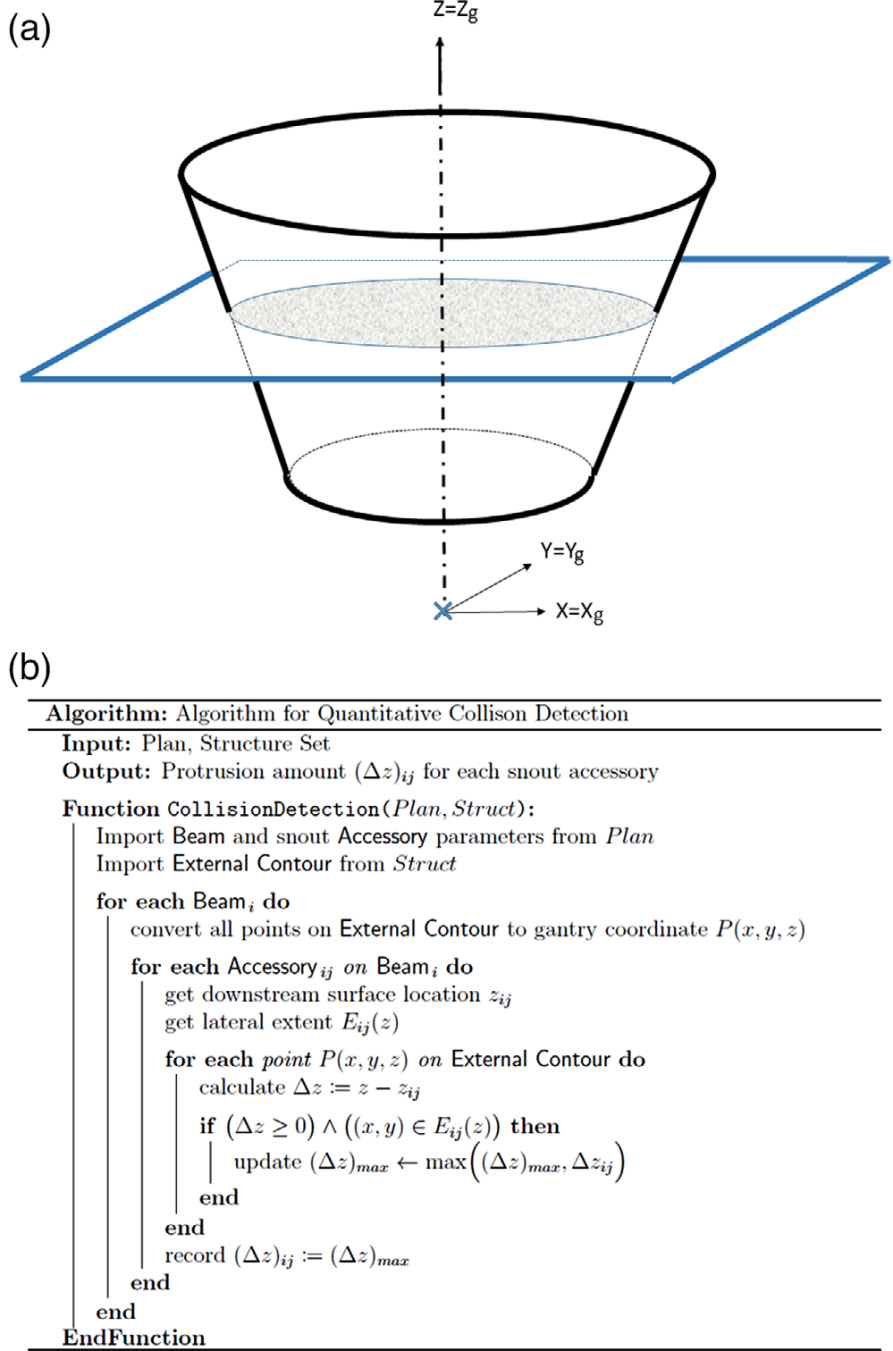
Schematic diagram (a) and pseudocode (b) for the automatic collision detection algorithm. All coordinates are in IEC gantry coordinate. IEC, International Electrotechnique Commission.

The framework utilizes the International Electrotechnique Commission (IEC) gantry coordinate system for collision detection. The collision detection is performed per beam where all points on the patient's contour are transformed into the specific beam's IEC gantry coordinates. Depending on the snout design, the snout accessories checked include range shifters, mounting apparatus for the range shifters such as the knob switch, snout casing, and nozzle covering. For simple three‐dimensional (3D) objects such as columns and cuboids with constant cross‐sections along the central axis, collisions can be detected simply by converting patient contour coordinates into the IEC gantry coordinate system and identifying those points that fall within the space occupied by the 3D object. For example, for a column shaped range shifter that is centrally mounted on the beam axis, any external contour point that is located above the downstream surface of the column and closer to the beam axis than the radius of the column, will cause a collision. The same principle applies for more complex 3D shapes like cone and pyramid frustums. The schematic diagram in Figure 1a illustrates the principle of detection and the pseudocode for the algorithm is given in Figure 1b. Details of this algorithm are described below. The program is implement with Matlab (Mathworks, Natick, MA, USA).

### 2.1 Snouts and their accessories

The various snout components are modeled by simple geometric shapes that best represent the 3D space they occupy. These 3D models simulate the relative positioning of the snout components with respect to the patient for potential collision detection. Only components that have caused collisions with the patient in our clinics are included in the model. Typically, collision is caused by the component that is most downstream of the snout, and for the majority of snout designs, it is the range shifter. In addition, the snout's external casing and other accessories, such as the range shifter's mounting apparatus, are also included due to collision with the patient under various treatment geometries.

### 2.2 External contour

The external contour is defined by a series of points on each slice of the planning CT. All points on all slices of the external contour are examined for potential collision. Note that if there exist any straight segments in the external contour, such as the outline along the flat bottom surface of the treatment table, only two points in space are typically used by the TPS to represent that straight section, that is, only one beginning point and one ending point along the straight section. However, collisions can still happen in the middle of the straight section where there is no explicit contour point. In order to ensure that these straight sections on the external contour are included for collision detection, additional points are inserted by linear interpolation to 0.5 cm between any two contour points that are greater than 2 cm apart.

### 2.3 Analytic algorithm for collision detection

The IEC gantry coordinate system is used in this framework for automatic collision detection.^41^ Briefly, the rotational axis of the gantry is defined as *+y*, the beam axis toward the radiation source is defined as *+z*, and *+x* is defined according to the right‐hand rule. Note that the IEC gantry coordinate system follows the gantry rotation and it coincides with the IEC fixed coordinate system at zero gantry angle. All nozzle and snout components are positioned in the IEC gantry coordinate relative to the isocenter. The external contour on the other hand is first converted from the DICOM standard Reference Coordinate System (RCS), that is, “patient coordinate”, to the IEC fixed coordinate, and later to the IEC gantry coordinate for each beam. The framework then examines each beam for potential collision. Specifically, the external contour is first translated to the isocenter of the beam under examination. For non‐zero table rotation, the external contour will additionally be rotated with respect to the isocenter in the IEC fixed coordinate before being converted to the IEC gantry coordinate. Note that for patients treated in a sitting position, an additional table pitch angle is applied before table rotation.

### 2.4 Coordinate transformation

Points on the external contour are in the patient coordinate $\left(x_{p},\;y_{p},\;z_{p}\right)$, where the subscript *p* denotes the patient coordinate. This patient coordinate is derived from DICOM standard RCS with the origin reset to the isocenter of each treatment fields, that is, with the isocenter coordinate values subtracted from the RCS coordinate values. To transform them into the IEC gantry coordinate, these points are first converted to the IEC fixed coordinate based on the plan orientation. This coordinate transformation can be expressed as $M_{fp}=M_{f}\;\times M_{p}$, where $M_{f}=\left[\begin{array}{ccc} 1 & 0 & 0\\ 0 & 0 & 1\\ 0 & -1 & 0 \end{array}\right]$ and $M_{p}$ is the plan orientation. Several commonly seen plan orientations such as head‐first spine (HFS), feet‐first supine (FFS), head‐first prone (HFP), and feet‐first prone (FFP) are given by $\left[\begin{array}{ccc} 1 & 0 & 0\\ 0 & 1 & 0\\ 0 & 0 & 1 \end{array}\right]$, $\left[\begin{array}{ccc} -1 & 0 & 0\\ 0 & 1 & 0\\ 0 & 0 & -1 \end{array}\right]$, $\left[\begin{array}{ccc} -1 & 0 & 0\\ 0 & -1 & 0\\ 0 & 0 & 1 \end{array}\right]$, $\left[\begin{array}{ccc} 1 & 0 & 0\\ 0 & -1 & 0\\ 0 & 0 & -1 \end{array}\right]$, respectively. Note that $M_{p}$ effectively converts $\left(x_{p},\;y_{p},\;z_{p}\right)$ from the patient coordinate to a coordinate system that is aligned with the HFS at zero table angle, and $M_{f}$ subsequently converts it to the IEC fixed coordinate defined above. For treatments performed in the sitting position, an additional matrix transformation $M_{\varphi }=\left[\begin{array}{ccc} 1 & 0 & 0\\ 0 & \cos\varphi & -\sin\varphi \\ 0 & \sin\varphi & \cos\varphi \end{array}\right]$ is needed to account for the pitch angle φ of the chair. Note that $M_{\varphi }$ reduces to the identity matrix when the pitch angle φ is zero. Table rotation θ is taken into consideration by $M_{\theta }=\left[\begin{array}{ccc} \cos\theta & -\sin\theta & 0\\ \sin\theta & \cos\theta & 0\\ 0 & 0 & 1 \end{array}\right]$ in the IEC fixed coordinate. Conversion from IEC fixed to IEC gantry coordinate follows the beam's gantry angle ϕ by $M_{\phi }=\left[\begin{array}{ccc} \cos\phi & 0 & -\sin\phi \\ 0 & 1 & 0\\ \sin\phi & 0 & \cos\phi \end{array}\right]$. Note that instead of rotating the gantry here we have effectively rotated the patient by −ϕ degrees into the IEC gantry coordinate. Overall, all points $\left(x_{p},\;y_{p},\;z_{p}\right)$ on the external contour are converted to the particular beam's IEC gantry coordinate $\left(x_{g},\;y_{g},\;z_{g}\right)$ by

$$\left(\begin{array}{c} x_{g}\\ y_{g}\\ z_{g} \end{array}\right)=M_{\phi }\times M_{\theta }\times M_{\varphi }\times M_{fp}\;\times \left(\begin{array}{c} x_{p}\\ y_{p}\\ z_{p} \end{array}\right)$$

Note that we have use the subscript *g* to denote the IEC gantry coordinate system.

### 2.5 Collision detection: Constant cross‐section (columns and cuboids)

Collision detection is done by comparing the external contour with the various snout components in the IEC gantry coordinate. For simplicity, the subscript *g* is dropped hereafter with the understanding that all coordinate values (x,y,z)are in the IEC gantry coordinate. It is also understood that hereinafter IEC gantry coordinate is referred to simply as gantry coordinate. As a first step, the *z* coordinate of all points on the external contour is checked to determine if it is above the downstream (i.e., closest to patient) surface of any of the snout accessories. If yes with respect to any snout component, an additional check is performed to ascertain whether its lateral extent (x,y)indeed resides inside the snout component. For column‐shaped range shifters, it's simply checking the radial distance from the central axis. Similarly for cuboid‐shaped range shifters, it's checking if |x| and |y| are both smaller than the half‐length and half‐width of the range shifter, respectively. For components that are mounted off the beam axis, such as the knob switch on the IBA (Ion Beam Applications, Walloon Brabant, Belgium) universal nozzle and the range shifter's mounting apparatus on the IBA dedicated nozzle, the extent of their lateral offsets is modeled accordingly.

### 2.6 Collision detection: Variable cross‐section (frustums)

For components that are not shaped as columns or cuboids, such as those shaped as cone or pyramid frustums, the same principle applies for collision detection. Specifically, all points on the patient's contour are again checked against the downstream surface of the snout accessories using their *z* coordinates. For any point (x,y,z) on the external contour whose *z* coordinate value is larger than that of the frustum's downstream surface, the difference in their *z* coordinates is used to infer the lateral extent of the frustum at the level of (x,y,z), as seen in Figure 1a. To determine if a point protrudes into the frustum, its lateral position (x,y) is checked against the lateral extent of the frustum at *z*. For a cone frustum centered to the beam axis, it's simply comparing the point's radial distance $\sqrt{x^{2}+y^{2}}$ with the frustum's radius at *z*. Note that the lateral extent of the frustum varies along the beam axis as a function of *z* and can be obtained by interpolating the dimensions of the upstream and downstream surfaces. Similar process applies to the pyramid frustum.

### 2.7 Quantitative collision clearance calculation

The framework also quantitatively determines the amount of snout retraction needed to avert a collision when it is detected. Without a collision, the framework will report the size of the air gaps for each snout component on a given beam. The *z* coordinate of its most downstream surface $Z_{c}$ is determined from the beam's snout extension and the accessory's dimensions. The differenceΔz in the *z* coordinates between each point (x,y,z) on the external contour and $Z_{c}$, that is, $\Delta z\;=Z_{c}\;-z$, is then determined. For any Δz≤0, the corresponding lateral coordinates (x,y)are used to determine if a collision indeed occurs, and all points that cause collision are recorded. The collision that has the most negative Δz, that is, the largest |Δz| value, has the largest protrusion and is therefore used to report the amount of snout extraction needed to avert collisions with the examined snout component. For all contour points withΔz>0, the lateral coordinates (x,y)are also used to determine if it is located within the lateral extent of the accessory's most downstream surface. This Δz value is recorded for air gap calculation. For snout components whose Δz values are all greater than zero for each point on the external contour, no collision occurs and the smallest Δz value is reported. Note that the smallest of these Δz values amongst all snout components is the air gap for the beam. In summary, the framework reports Δz>0 values to indicate available clearance and Δz≤0 values to indicate collisions.

### 2.8 Different snout designs and their models

**FIGURE 2:**
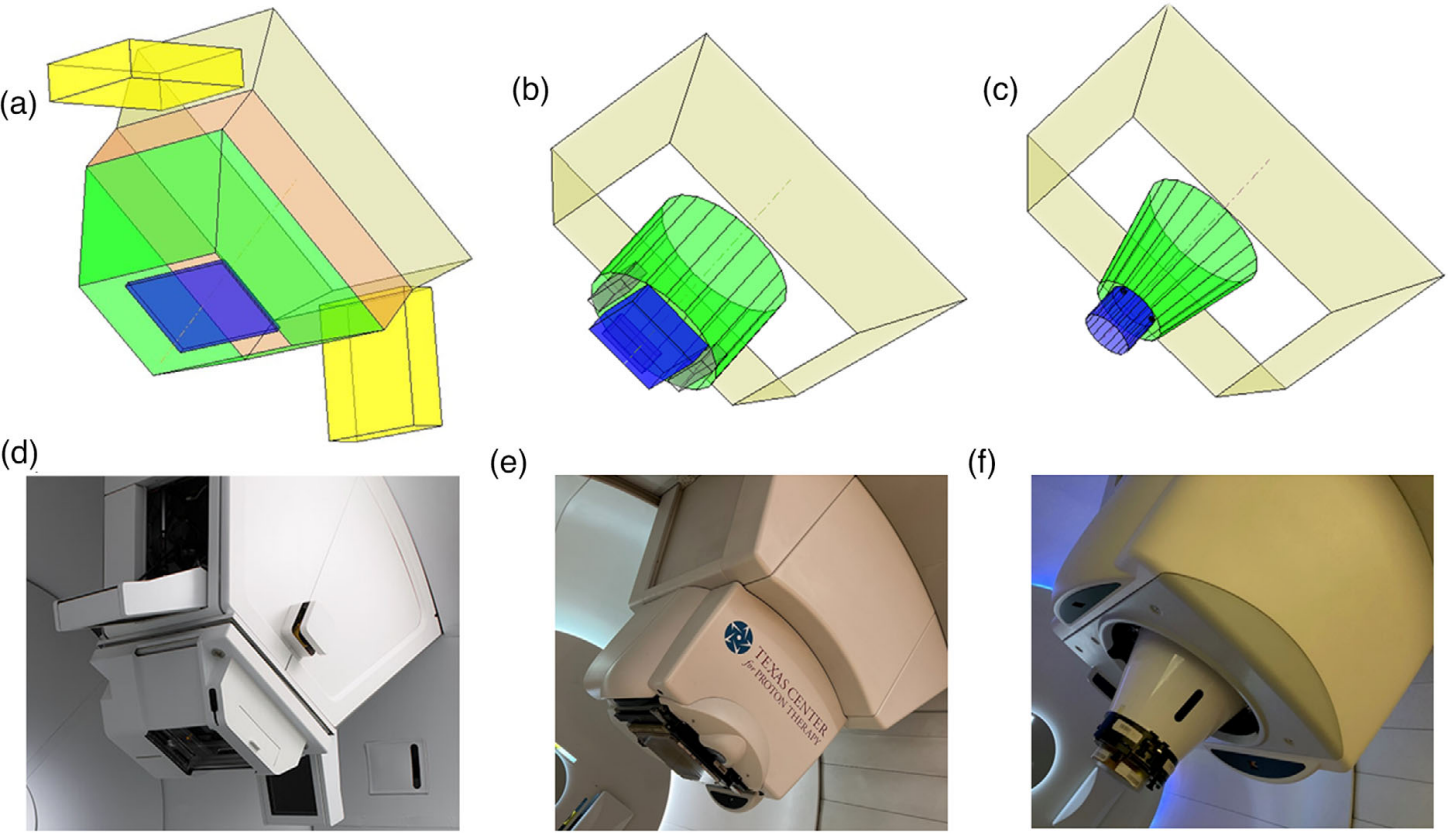
Different snout designs and their models in the framework: Varian (a, d), IBA dedicated (b, e), and IBA Universal (c, f).

Figure 2 shows three typical snout designs and their corresponding models in this framework. The range shifters are modeled by columns or cuboids (blue). The snout casings are either cone or pyramid frustums (green). The nozzle, which is upstream of the snout, is modeled as a pyramid frustum (beige).

For the Varian (Varian Medical Systems, Palo Alto, CA) nozzle shown in Figure 2a,d, the downstream surface of the pyramid frustum that models the snout's casing is monitored for collision detection since the range shifters are enclosed within the snout. The framework still includes the range shifter here in order to visualize the 2 cm inward offset of the range shifter from the bottom of the snout. This space can potentially be exploited as an extra margin against potential collision since the snout is open at its downstream surface (Figure 2a,d). Note that this offset is identical for all range shifters regardless of their thickness. For the purpose of collision detection, an alert will still be produced if the external contour protrudes into the space between the bottom of the snout and the range shifter.

The dedicated nozzles from IBA are for PBS only and this type of nozzle houses the range shifter exteriorly downstream of the snout (Figure 2e). The snout moves the range shifter along the central axis to the designed location. Here the range shifter is modeled as a movable cuboid along the beam axis. Note that the mounting apparatus for the range shifters is modeled here by three cuboids (black) arranged in a horseshoe formation as seen in Figure 2b.

IBA's universal nozzle, as seen in Figure 2c,f, is designed to work with both PBS and DS, and its snout can accommodate both patient‐specific apertures and range shifters. Patient‐specific apertures are positioned upstream of the range shifter, enclosed entirely within the snout. As a result, patient‐specific apertures have not caused collisions in the clinic, and only range shifters are included here. Nevertheless, snout accessories such as the knob‐shaped switches that clinched the range shifter in place onto the snout, are included. These knobs are located off‐axis to the side of the range shifter. Although they are small and slightly upstream of the range shifter's most downstream surface, these knobs have caused collisions in the clinics, especially for beams with couch kicks. Such collision is unlikely to be caught during planning since the knobs are not specifically modeled within the TPS.

## 3 RESULTS

### 3.1 Complex treatment geometries

**FIGURE 3:**
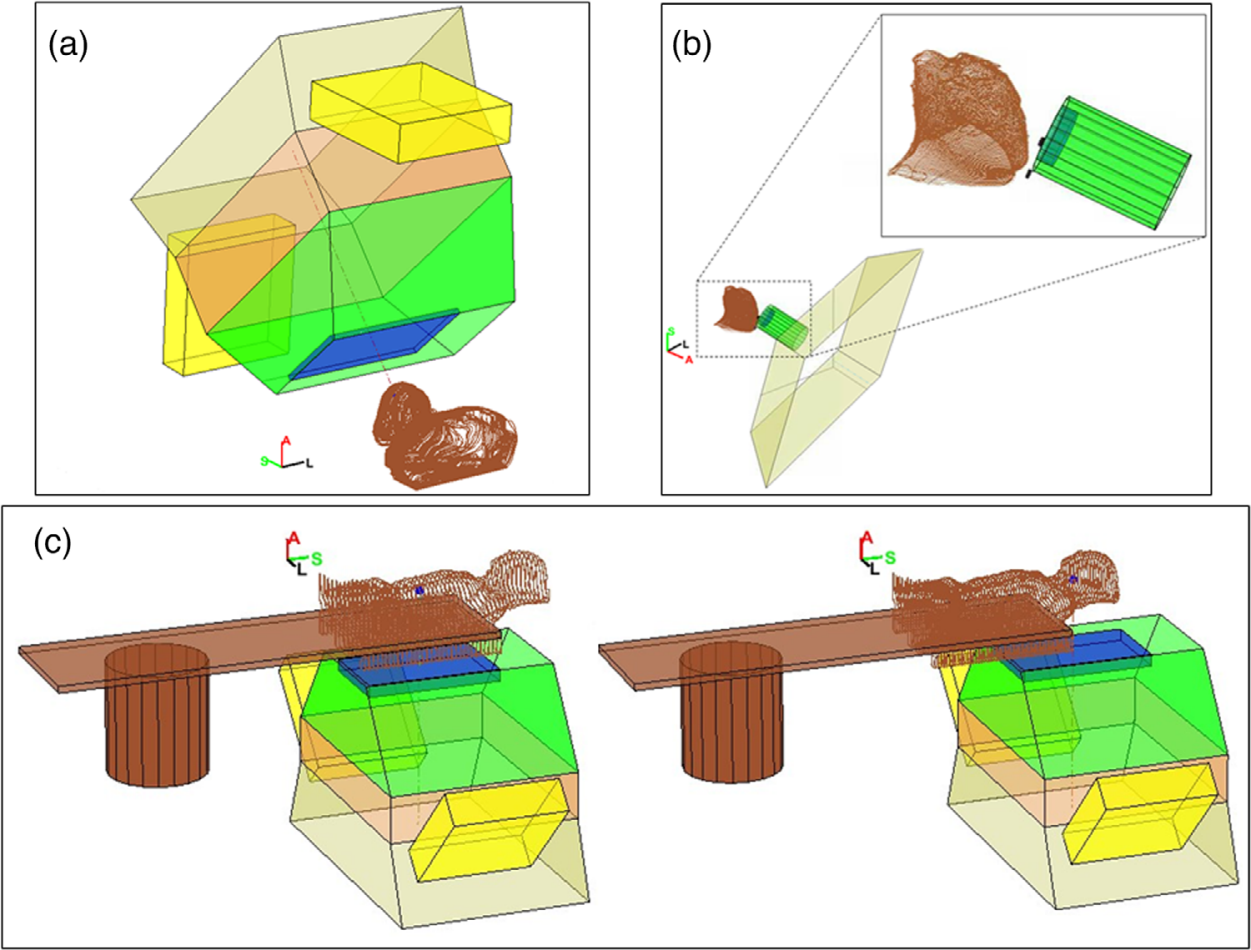
Complex treatment geometries modeled by the framework: Non‐coplanar beam arrangement (a), chaired treatment position (b), and multiple isocenter treatment (c).

The framework is capable of modeling couch kicks (Figure 3a), patients in a treatment chair (Figure 3b), and multiple‐isocenter plans (Figure 3c). Figure 3a demonstrates a typical brain case where a near‐vertex beam is used to treat the superficial lesion. The table is angled at 90 degrees with the gantry at 320 degrees to avoid the brainstem. Note here for this particular snout design, the range shifter is centered with respect to the central axis but the overall snout is off‐centered in the *Y* direction (IEC gantry coordinates) to accommodate sensors and electronics. A patient in the sitting position is shown in Figure 3b where the patient orientation is FFS with a table pitch of −70 degrees. Table rotation angle is at 250 degrees and gantry angle for this particular beam is at 90 degrees. A cranial‐spinal irradiation (CSI) case is shown in Figure 3c, where two posterior‐anterior beams have different isocenter coordinates for the superior and inferior portions of the target, respectively.

### 3.2 Collision aversion in the clinic

**FIGURE 4:**
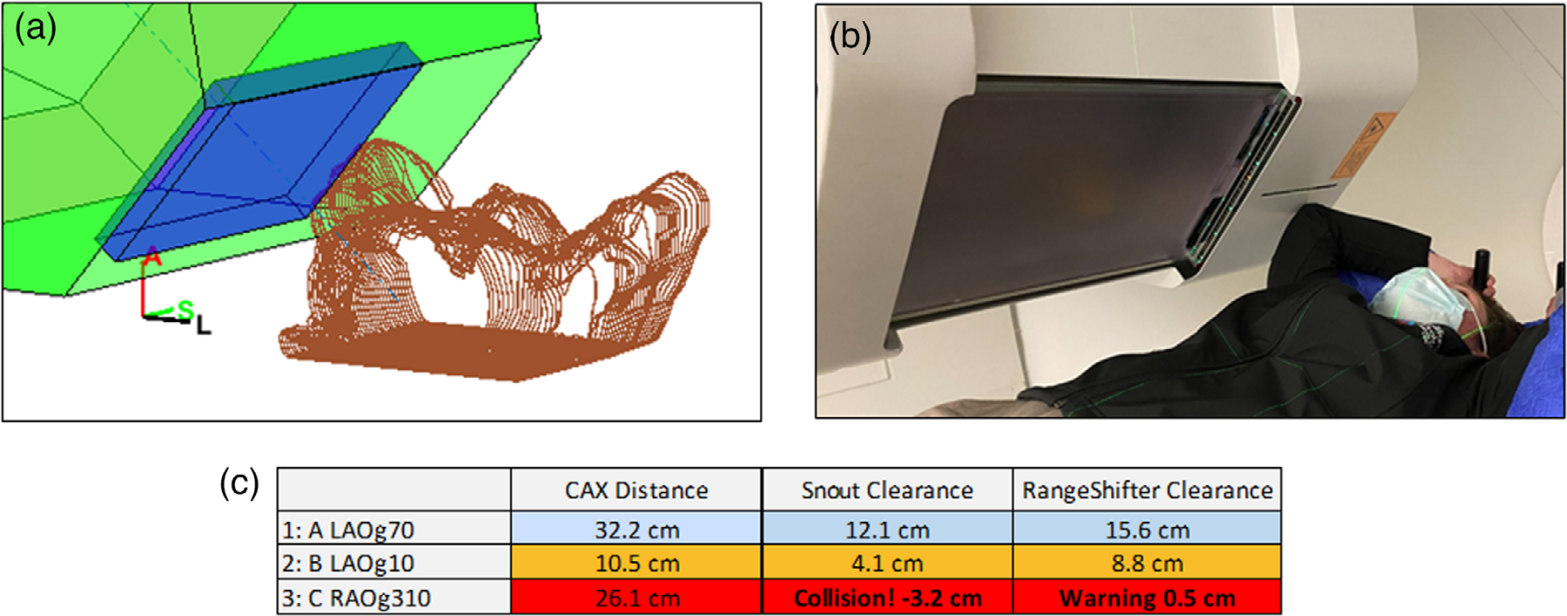
Collision between the patient's elbow and the snout casing, as seen in the model (a) and in the treatment room (b). A collision alert (red) was flagged for collision (clearance less than zero) for the RAO beam (c, third row), and a warning (orange) is displayed for minimum clearance less than 5 cm for the LAO beam at gantry 10o (c, second row). The other LAO beam at gantry 70o is cleared (c, first row).

Figure 4 demonstrates a clinical case where a potential collision was averted using this framework. Typical immobilization for proton breast treatments requires patients to have their arms up. To minimize the lateral penumbra for the shallow target, the planners typically move the range shifter as close to the chest as possible. However, in this case during planning, the snout was calculated to collide with the patient's elbow on the ipsilateral side for the right‐anterior‐oblique (RAO) beam, as seen in Figure 4a. Following the quantitative snout retraction value recommended by the framework, the planner was able to proceed with planning without needing to simulate a patient setup in the treatment room. As seen in Figure 4c, the RAO beam is shown to have a negative distance of 3.2 cm (shown in red), that is, the patient's elbow protrudes into the snout casing by 3.2 cm (Figure 4a,b).

### 3.3 Dosimetric effect

**FIGURE 5:**
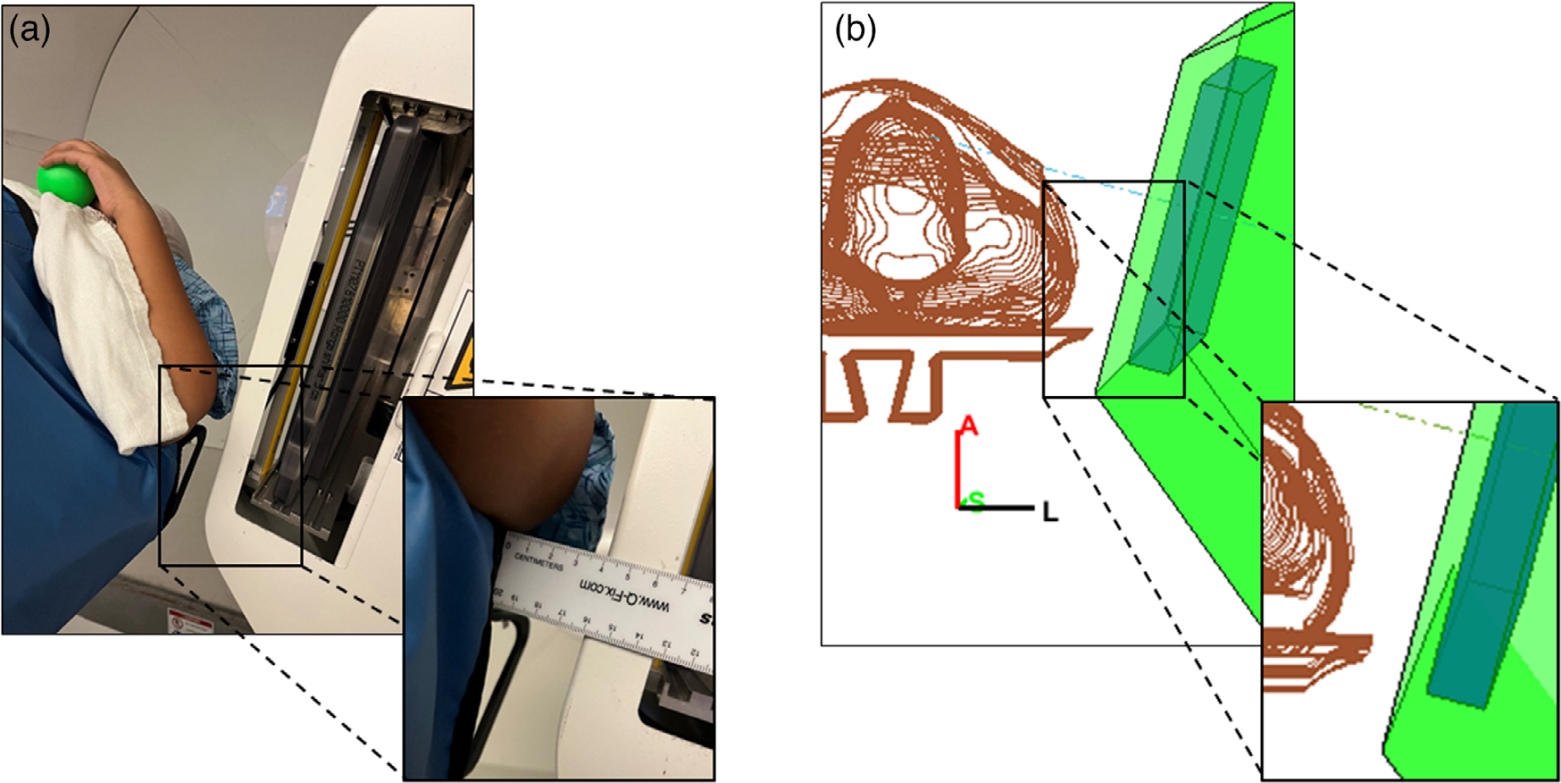
Treatment geometry for an orbital rhabdomyosarcoma case as seen in the treatment room (a) and the framework model (b). The minimum clearance measured on the first day of treatment is 4 cm and the framework's prediction is 3.7 cm, demonstrating that the framework's model accuracy is within fractional setup uncertainty.

**FIGURE 6:**
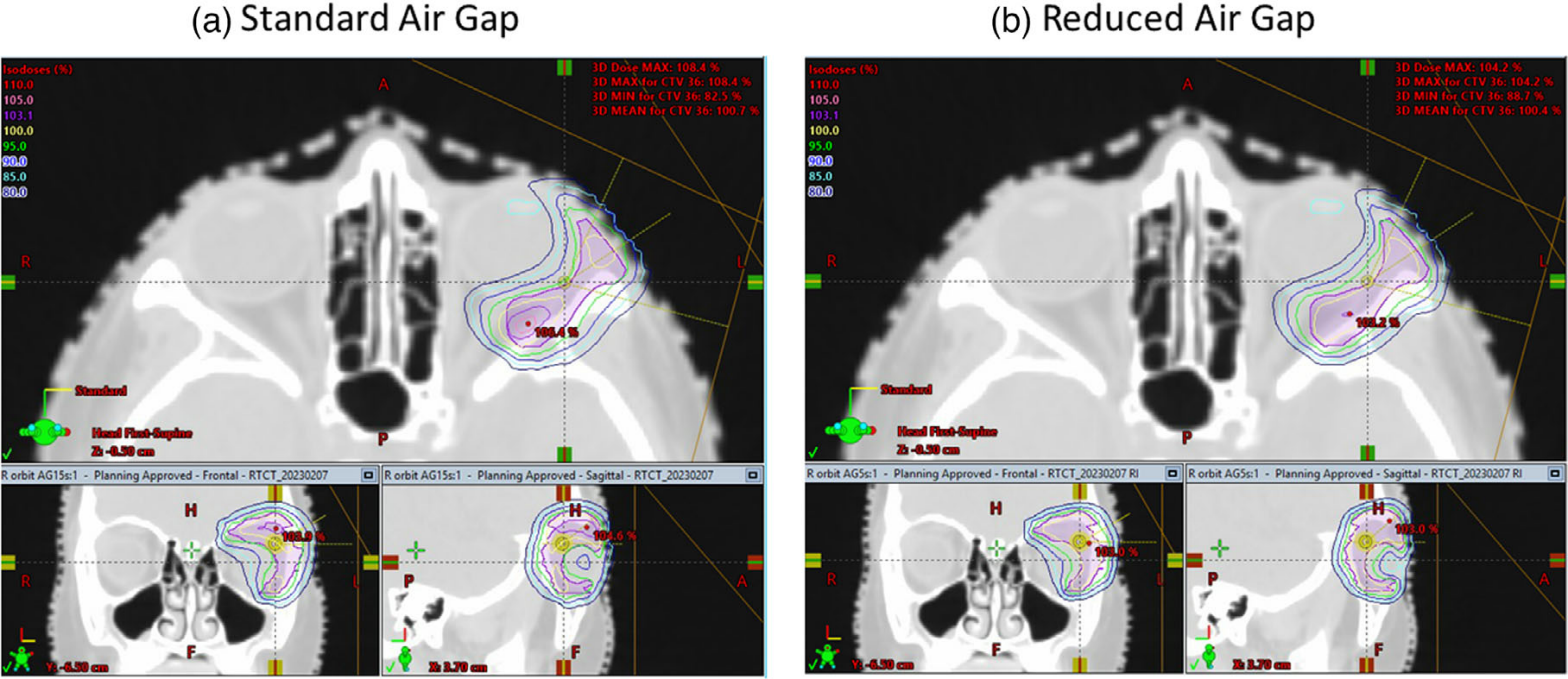
Dosimetric comparison between plans with standard (a) and reduced (b) air gaps for the orbital rhabdomyosarcoma case. The plan with reduced air gap is more conform with lower dose to the lens.

Prior to the clinical implementation of this framework, our clinic used a standard air gap of 15 cm for all beams that require a range shifter in order avoid collisions. Here an orbital rhabdomyosarcoma case is examined to demonstrate the dosimetric benefit of reducing the air gap. The collision detection framework enabled reduction of the air gaps from 15 cm to between 5 and 8 cm for the three beams in this case. The plan was then re‐optimized using the same dose‐volume criteria. As seen in Figure 5, the framework predicts that the left‐posterior oblique beam has a clearance of 3.7 cm between the snout and the treatment table, and the actual measurement on the first day of treatment is 4.0 cm. This difference of 3 mm is within the range of inter‐fractional setup variations. Figure 6 shows the dosimetric comparison between plans using standard and reduced air gaps. It is clear that the penumbra (95% to 80%) reduced from 6 mm with the standard air gap to 4 mm with the reduced air gap. The mean doses to the left lens and the left globe was also reduced from 18.4 to 14.5 Gy, and 26.3 to 25.0 Gy, respectively. In addition, the plan with reduced air gap is more conformal with its overall Dmax at 104.2%, as opposed to the Dmax of 108.4% for the standard air gap, given the same CTV coverage of V95% = 99%. Here, the smaller air gap reduced lateral penumbra and allowed the optimizer to conform better with the target anatomy, demonstrating the framework's capability to accurately model treatment geometry and enabled better plan dosimetry.

### 3.4 Validation on accuracy

A validation study on the model's accuracy was performed using a pelvis anthropomorphic phantom. The phantom was scanned head‐first supine and a target contour was drawn on the left pelvic bone. A plan with four beams was generated at orthogonal gantry angles, 0°, 90°, 180°, and 270°. The phantom was then aligned in the treatment room using standard kV image‐guided radiation therapy. Distance from the bottom of the snout to the closest point on either the phantom or treatment table was measured for each beam. For the AP beam at gantry 0°, the closest point is on the phantom. For the other three beams, the closes point is on the edges of the table (90° and 270°) and the rails under the table (180°). Results of this validation is shown in Table 1. The maximum deviation between the model's prediction and the measurements is 8 mm at gantry 270°. Deviations at Gantry 0°, 90°, and 180° are 5, 4, and 0 mm, respectively.

**TABLE 1:** Validation on the accuracy of the model.

	Model prediction	Measurement
AP Gantry 0°	14.0 cm	14.5 cm
LL Gantry 90°	13.9 cm	13.5 cm
PA Gantry 180°	14.0 cm	14.0 cm
RL Gantry 270°	10.3 cm	9.5 cm

### 3.5 Calculation speed

Speed of the program was evaluated using a total of 12 plans. The computer uses Windows Server 2012 Standard on an Intel Xeon 3.4 GHz 4‐core CPU with 32 GB RAM. On average, the calculation time for automatic collision detection is about 0.16 s. The average read time to load the DICOM plan file is 0.59 s, and to load the DICOM structure set file takes about 5.89 s. Currently these DICOM file read times are required because the framework is built on a stand‐alone system, that is, not part of the TPS. One would need to export the treatment plan and the structure set as DICOM files from the TPS in order for the framework to import and analyze for potential collisions. We envision integrating this framework into commercial TPS's using their application programming interface (API), and eliminating the steps needed for exporting and importing DICOM files. In addition to collision detection, the program also performs the other functionalities and their average calculation time are as follows: (1) checking the correctness of the field names against actual gantry and table angles, 0.06 s; (2) plotting the gantry and the various snout accessories, 0.10 s; and (3) plotting the patient's external contour, 0.44 s. Results of the timing test is summarized in Table 2.

**TABLE 2:** Evaluation on the model's calculation speed.

	CSI	Liver	Left Orbit	Left Breast	Rib	Bilat HN	Brain	HN	Right Breast 1	Right Breast 2	Right lung	Prostate pelvis	Avg
Load DICOM plan	0.90	0.36	0.56	0.44	0.32	2.03	0.77	1.30	0.51	0.41	1.35	0.28	0.59
Load DICOM struct	8.65	0.48	4.76	4.49	3.49	17.87	15.28	12.58	1.19	0.85	2.73	3.13	5.89
Collision Calc	0.10	0.03	0.14	0.15	0.04	0.05	0.07	0.12	0.05	0.12	0.14	0.21	0.16
Beam name check	0.05	0.02	0.21	0.05	0.20	0.26	0.05	0.04	0.04	0.03	0.07	0.08	0.06
Plot snout	0.10	0.10	0.11	0.12	0.11	0.13	0.13	0.10	0.11	0.11	0.13	0.11	0.10
Plot patient	0.38	0.16	0.33	0.16	0.13	0.26	0.33	0.36	0.25	0.17	0.78	0.49	0.44

## 4 DISCUSSION

Snouts with protruding mounting apparatus are designed to allow the range shifters to be placed closer to the patient, resulting in smaller air gaps and tighter lateral penumbra. However, while such snout designs enable better dosimetry, it is more difficult to predict potential collisions at the time of planning. For snout designs whose range shifters are enclosed interiorly, one would only need to consider the snout's outer casing for potential collisions. Nevertheless, this type of snout is in general larger in size, and hence has an increased risk for potential collisions.

Accurate representation of the patient, treatment table and immobilization devices by the external contour is required for the accurate detection of potential collisions. Sections of the patient that are not included in the CT will not be used to substantiate the external contour, and as a result, will not be taken into consideration by the framework for collision detection. Since the CT scan taken for radiation therapy is typically limited only to the treatment site, collisions outside the scope of the CT scan can still occur. To avoid potential collisions evading the framework, one should extend the scanned region. Using the breast case above as an example, for all cases where the patients are immobilized with their arms up, the planning CT would be extended to include the elbow as a standard of practice in our clinic.

**FIGURE 7:**
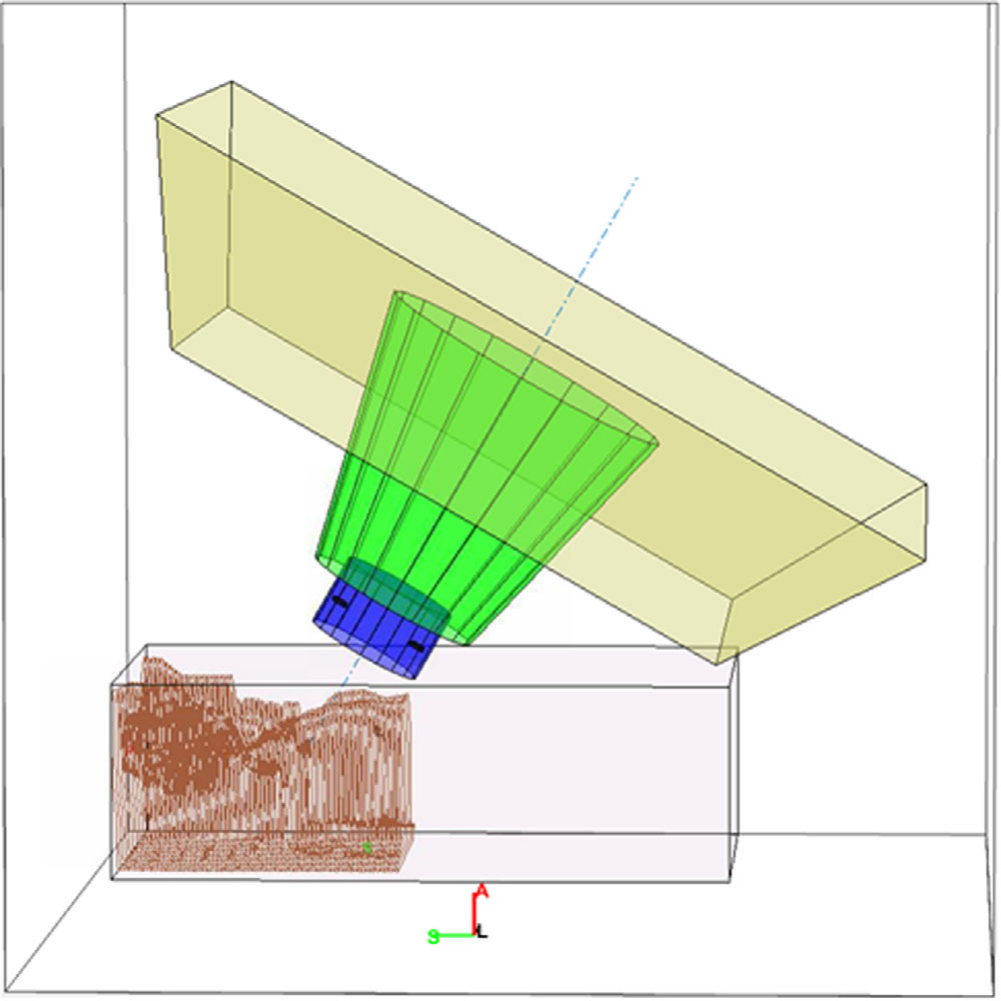
A bounding box is used to estimate the extent of the patient for a mediastinum case. The beam angle is selected to minimize radiation to the uninvolved breasts.

When an extended CT is not available, an alternative method to address this issue is to use a bounding box to approximate the patient. As seen in Figure 7, a bounding box is generated to encapsulate the patient where the most anterior, posterior, left, right, superior, and inferior coordinates of the external contour are used to defined the six bounding surfaces. Values of these coordinates are also shown on the graphical user interface as three sets of two‐tuples, representing posterior(+)/anterior (−), left(+)/right(−), and superior(+)/inferior(−). These values are measured relative to the isocenter and users can change the size of the bounding box by entering new values. For example, to display the full height of the patient, the planner would enter the distances from the isocenter to the top of the head and to the bottom of the feet, respectively for the superior and inferior values. The bounding box can help planners visualize the approximate extent of the patient.

Since this analytic framework is capable of determining the minimum distance between the patient and the various snout components quickly for all gantry angles, it can be used to assure clearance for proton arc therapy. In addition, a constant minimum air gap, instead of a constant snout extension, at all gantry angles can potentially further improve the dosimetry for proton arc therapy. For current PBS plans with discrete beam angles, this functionality can also be used to advise concurrent gantry and snout movements between the various beams within a plan. With the framework's a priori quantitative knowledge of the minimum clearance needed at all gantry angles, concurrent movements of the gantry and the snout can reduce the amount of time patients stay on the treatment table, therefore improving patient comfort and throughput.

To the best of our knowledge, this framework is novel in that it performs quantitative collision detection using actual patient‐specific external contour with a simple analytic algorithm. The framework does not rely on a generic phantom for collision detection. In addition, the framework can take into considerations complex treatment geometries such as table kicks, chaired treatment position and multiple isocenter plans

### 4.1 Limitations

The current approach has limitation in that it can only model objects with surfaces perpendicular to the beam axis, that is, right frustums. Oblique frustums, such as physical wedges often seen on x‐ray linacs, would require additional steps in the algorithm. Using a physical wedge as an example, the algorithm would use a plane perpendicular to the beam axis to intersect the wedge. The extent of the intersection between the plane and the wedge will be defined by the plane's z coordinate in the gantry coordinate. Given any particular point $(x_{o},\;y_{o},z_{o})\;$on the patient's external contour in gantry coordinate, one would set the *z* coordinate of the interesting plane to be $z_{o}$, follow the same principle described in the framework to define the intersection with the wedge, and determine if $\left(x_{o},\;y_{o}\right)$ is inside the wedge. Algorithmically, each point on the patient's external contour will be converted to the gantry coordinate and each individual point's $z_{o}$ value will be used to formulate the plane perpendicular to the beam axis. The extent of the intersection in gantry coordinate's *x* and *y* directions is determined by the physical wedge's slant angle. By comparing this 2D extent with the point's $\left(x_{o},\;y_{o}\right)$, one can determine if the point is inside the wedge. This same principle would apply for irregularly shaped snout accessories as long as a cross‐sectional model of the accessory is available along the beam axis. For each point on the patient's external contour in the gantry coordinate, the *z* coordinate value will be used to generate a plane parallel to the beam axis. The extent of intersection can then be numerically determined and used to compare with the lateral position of the point. Additionally, for snout accessories positioned off the beam axis, one would offset these accessories’ *x* and *y* gantry coordinate values to account for their off‐axis placements, as demonstrated by the off‐axis racks and knobs seen in Figures 2e and 3b that are used to hold the range shifters in place on the snout. Both right and oblique frustums can be offset to off‐axis locations and modeled using the principle described above.

The robotic arm, on which the treatment table is attached, is not currently included into the framework. It could potentially collide with the nozzle's cover for posterior beams. In order to model the robotic arm properly, the patient's position on the treatment table must be accurately indexed.^27^ This requirement can be attained for cases where the immobilization device is indexed by a locating bar. For cases without an indexed immobilization, the relative position of the patient on the table can only be estimated. In addition, modeling the motion of the robotic arm as a function of table rotation and translation is also required. Further works on this framework would therefore include the incorporation of the robotic arm into the external contour.

## 5 CONCLUSION

An analytic framework was presented here that can simulate patient treatment position and automatically detect potential collisions by quantifying the exact amount of snout retraction needed to avert such collisions. This framework enabled the planners to utilize smaller air gaps for better dosimetry, and at the same time ensured that the planned treatment geometry is indeed realistically achievable in the treatment room.

## AUTHOR CONTRIBUTIONS

Stephen K. Northway coded and tested the program, Bailey M. Vallejo and Emily E. Hansen acquired and analyzed data, Lawrence Liu conducted the validation test, Shikui Tang and Dennis Mah acquired and analyzed data, Iain J. MacEwan and James J. Urbanic co‐conceptualized the project, Chang Chang co‐conceptualized the project, coded the first version of the program and drafted the manuscript. All authors contributed to the editing and revision of the manuscript.

## CONFLICT OF INTEREST STATEMENT

This study was partially funded by Varian Medical Systems.
